# Psychological Distress and Self‐Rated Health Among Single Fathers and Mothers During the COVID‐19 Pandemic in Japan

**DOI:** 10.1002/jgf2.70128

**Published:** 2026-05-24

**Authors:** Yasutaka Kuniyoshi, Akihito Uezato, Takahiro Tabuchi

**Affiliations:** ^1^ Department of Social Services and Healthcare Management International University of Health and Welfare Otawara Japan; ^2^ Center for Basic Medical Research International University of Health and Welfare Otawara Japan; ^3^ Center for Medical Innovation Institute of Science Tokyo Tokyo Japan; ^4^ Division of Epidemiology, School of Public Health Tohoku University Graduate School of Medicine Sendai Japan

**Keywords:** extended family, family structure, income, occupations, single parent

## Abstract

**Background:**

The COVID‐19 pandemic exacerbated health burdens, particularly for vulnerable populations. Single parents face unique preexisting challenges, and their health during the pandemic remains insufficiently investigated. We aimed to compare psychological distress and self‐rated health (SRH) between single and partnered parents during the pandemic, considering the influence of socioeconomic factors.

**Methods:**

We analyzed the data of 16,028 parents aged 18–50 years with children aged 14 years or younger from the Japan COVID‐19 and Society Internet Survey (JACSIS) 2020–2022. Psychological distress (Kessler K6 scale, ≥ 5) and SRH (very poor or poor) were compared between single (200 single fathers/1330 single mothers) and partnered parents using chi‐squared tests and multivariable logistic regression, adjusting for confounders. Furthermore, household structure, annual household income, occupation, and working hours were stratified for the analysis.

**Results:**

Across 2020–2022, psychological distress and poor SRH were significantly more prevalent among single parents than among partnered parents. Single parents also reported lower income and higher nonregular employment. These disparities persisted across the stratifications. Multivariable logistic regression confirmed higher adjusted odds ratios for psychological distress (single fathers: 1.50–2.18; single mothers: 1.23–1.66) and poor SRH (single fathers: 0.74–2.93; single mothers: 1.26–1.79) associated with single‐parent status.

**Conclusions:**

During the COVID‐19 pandemic, single parents in Japan experienced greater psychological distress and poorer SRH than partnered ones, highlighting their heightened vulnerability. Therefore, targeted support, particularly addressing economic hardship, is crucial to mitigate these health disparities and improve the resilience of single‐parent households during public health emergencies.

## 1 Introduction

The COVID‐19 pandemic has profoundly affected societies worldwide, causing economic disruptions [1], social isolation [2], and increased psychological distress [3, 4, 5]. The pandemic has adversely affected both mental health [4] and physical well‐being [6] in the general population, with notable increases in anxiety, depression, and stress‐related disorders. These health burdens have been pronounced among socially and economically vulnerable groups, especially single parents and low‐income populations [3].

Even before the pandemic, single parents consistently faced significant challenges, including financial insecurity, limited childcare support, demanding caregiving responsibilities, and unstable employment conditions, all contributing to the increase in the risks of developing poorer mental and physical health [7, 8, 9, 10, 11, 12]. The pandemic likely intensified these preexisting stressors. Indeed, several international studies have highlighted the profound health impacts of the COVID‐19 crisis and the corresponding psychological distress experienced by single parents [13, 14]. However, the specific impacts within Japan remain insufficiently investigated, despite the Japanese single‐parent households' distinct structural vulnerabilities. Among Organization for Economic Co‐operation and Development (OECD) countries, Japan has one of the highest employment rates for single mothers, but it also paradoxically records one of the highest relative poverty rates for this population because of the prevalence of low‐paying, nonregular employment [15]. Prepandemic studies in Japan have reported that psychological distress is highly prevalent among both single mothers and single fathers, stemming from financial hardship and work–family conflicts [16, 17]. Since the COVID‐19 pandemic, domestic studies have begun to explore the specific stressors and coping strategies of Japanese single mothers [18]. Nevertheless, the impact of the pandemic on the well‐being of both single fathers and mothers, considering key socioeconomic moderators such as multigenerational living arrangements and income, remains unclear.

Therefore, utilizing data from the large‐scale Japan COVID‐19 and Society Internet Survey (JACSIS; https://jacsis‐study.jp/), spanning three pandemic years (2020–2022), this study primarily aimed to determine the effects of the prolonged COVID‐19 crisis on the mental and physical health of single parents in Japan compared with partnered parents. On the basis of their preexisting vulnerabilities, which were likely magnified by pandemic‐specific stressors (increased social isolation and employment insecurity), we hypothesized that single parents exhibited significantly greater psychological distress and poorer SRH than partnered parents during the pandemic. Another hypothesis is that these amplified health disparities are highly associated with underlying socioeconomic factors, particularly low household income and unstable employment, which were severely exacerbated by the economic fallout during the COVID‐19 crisis. Additionally, we explored whether these adverse health outcomes were mitigated by living in a multigenerational household, traditionally considered a protective factor but potentially a source of infection anxiety and caregiving burden during the pandemic. Finally, we longitudinally assessed temporal trends in health outcomes among a subset of participants as a post hoc exploratory analysis to understand the potential cumulative burden over the prolonged 3‐year pandemic period. Elucidating these associations may provide evidence crucial for developing targeted policies and interventions to support the health and well‐being of single‐parent households in future public health crises.

## 2 Methods

### 2.1 Study Design and Data Source

This study used data from JACSIS, which was conducted annually from 2020 to 2022. Data were analyzed separately for each survey year to explore potential temporal trends in health outcomes. JACSIS is a large‐scale, web‐based survey designed to evaluate the pandemic's social and health impacts in Japan. Data were collected using an internet‐based self‐reported questionnaire administered to a large panel of Japanese adults. Participants were recruited through Rakuten Insight Inc. (Tokyo, Japan), a leading internet survey company that manages Japan's largest single survey panel, comprising approximately 2.2 million members across all prefectures as of September 2022. In total, 29,000, 31,000, and 32,000 participants responded in surveys 2020 (August–October 2020), 2021 (September–October 2021), and 2022 (September–October 2022), respectively. In particular, 28,000 individuals from the general population and 1000 single parents participated in survey 2020.

All procedures conformed to the ethical standards of the Helsinki Declaration of 1975, as revised in 2013. Research Ethics Committee of the Osaka International Cancer Institute reviewed and approved the survey protocol (approved on June 19, 2020; approval number: 20084‐2). Additionally, this study was reviewed and approved by the Ethical Committee of the International University of Health and Welfare (No. 24‐TC‐031). The internet survey agency respected the Act on the Protection of Personal Information in Japan. Before responding to the online questionnaire, all participants provided web‐based informed consent. A credit point known as “Epoints,” which could be used for internet shopping and cash conversion, was provided to the participants as an incentive. Furthermore, this study was conducted according to the Strengthening the Reporting of Observational Studies in Epidemiology Statement [19].

### 2.2 Study Population

To ensure data quality, we excluded respondents showing discrepancies and/or artificial/unnatural responses particularly to the following three questions: (i) “Please choose the second from the bottom” (providing an invalid response to the attention check question); (ii) “Please choose positive in all questions for using drugs,” (selecting positive responses to all questions about drug use [marijuana, cocaine, heroin, etc.]); and (iii) “Please choose positive in all questions for having chronic diseases” (selecting positive responses to all questions about alternative chronic conditions).

Conversely, adults aged 18–50 years who had at least one child aged 14 years or younger were included. The family type was categorized into two groups: single‐parent and partnered‐parent households. The participants were stratified by sex (father or mother). Ultimately, they were divided into four groups: single fathers, single mothers, partnered fathers, or partnered mothers.

Marital status was determined by the participant's response to the following question: “Are you currently married (including those living together as a couple without being legally married)?” Response options included: Married, Unmarried, Widowed, or Divorced. Thus, single parents include both divorced and bereaved individuals.

### 2.3 Measurement

#### 2.3.1 Outcome

##### 2.3.1.1 Psychological Distress

Psychological distress was measured using the six‐item Kessler Psychological Distress Scale (K6) [20]. This brief, validated screening tool has six items that assess the symptoms of anxiety and depression over the past 30 days, yielding a total score of 0–24. The higher the score, the greater the psychological distress is. For the analysis, we categorized the K6 scores into three groups, consistent with previous studies [21, 22]:

- Low distress: 0–4
- Moderate distress: 5–12
- High distress: 13–24

##### 2.3.1.2 Self‐Rated Health (SRH)

SRH was evaluated using a single question with a 5‐point Likert scale response (Excellent, Good, Fair, Poor, or Very Poor). Although simple, SRH is a robust and extensively validated measure globally, recognized as a powerful predictor of future health trajectories, including mortality and functional decline [23, 24]. Thus, this tool has become a highly efficient screening indicator for the overall health status. For the analysis, the responses were dichotomized into the following:

- “Not Poor” (Excellent, Good, and Fair)
- “Poor” (Poor and Very Poor)

#### 2.3.2 Covariates

The following demographic and socioeconomic variables were included as covariates: age (18–20, 21–30, 31–40, or 41–50 years), educational attainment (junior high school, high school/vocational school, university/junior college/technical college, or other), number of children aged below 14 years (1, 2, 3, or ≥ 4), household structure (three‐generation or nuclear family, determined by living with own parents or parents‐in‐law), annual household income (< 3, 3–10, > 10 million JPY, or no response/unknown), occupation (unemployed, nonregular employee, full‐time employee, self‐employed, employer), working hours (< 20, 20–40, 40–60, or ≥ 60 h per week), and medical conditions (yes or no for the following: cancer, noncancer physical illness, depression, or other mental disorders).

### 2.4 Statistical Analysis

Descriptive statistics were computed for all study variables, stratified by family type (single‐parent or partnered‐parent type) and sex (father or mother), resulting in four groups (single fathers, single mothers, partnered fathers, and partnered mothers). To examine group differences, we analyzed K6 and SRH across the following stratifications:

1. Sex, family type, and household structure.
2. Sex, family type, and household income.
3. Sex, family type, and occupation.
4. Sex, family type, and working hours.

Moreover, categorical variables between the groups were compared using the chi‐squared test.

We also employed logistic regression analyses to examine the associations between family type and health outcomes, adjusting for key covariates including age, educational attainment, number of children, and presence of each chronic disease (cancer, noncancer physical illness, depression, and other mental disorders). With reference to previous studies, we did not control for factors such as family structure, household income, employment, and working hours, which could be potential mediating factors. A complete‐case analysis was conducted, and individuals with missing data on any of the covariates included in the models were also excluded from the final analysis. No imputation methods were used.

To examine longitudinal trends, we analyzed a subset of participants (*N* = 1282) who completed all three surveys (2020–2022) and maintained a consistent family type (single‐parent or partnered‐parent type). This subset excluded 13 individuals (from 1295 participants who initially completed all the surveys) because of changes in the reported family type. In this cohort, the prevalence of health outcomes was calculated and stratified by family type. Changes in the proportions over the 3‐year period were assessed using Cochran's *Q* test.

All statistical data were analyzed using Python (version 3.11.11) on Google Collaboratory and R version 4.4.1 (R Foundation for Statistical Computing, Vienna, Austria). For the logistic regression analysis, we used the “glm” function in R.

## 3 Results

### 3.1 Participant Characteristics

**FIGURE 1:**
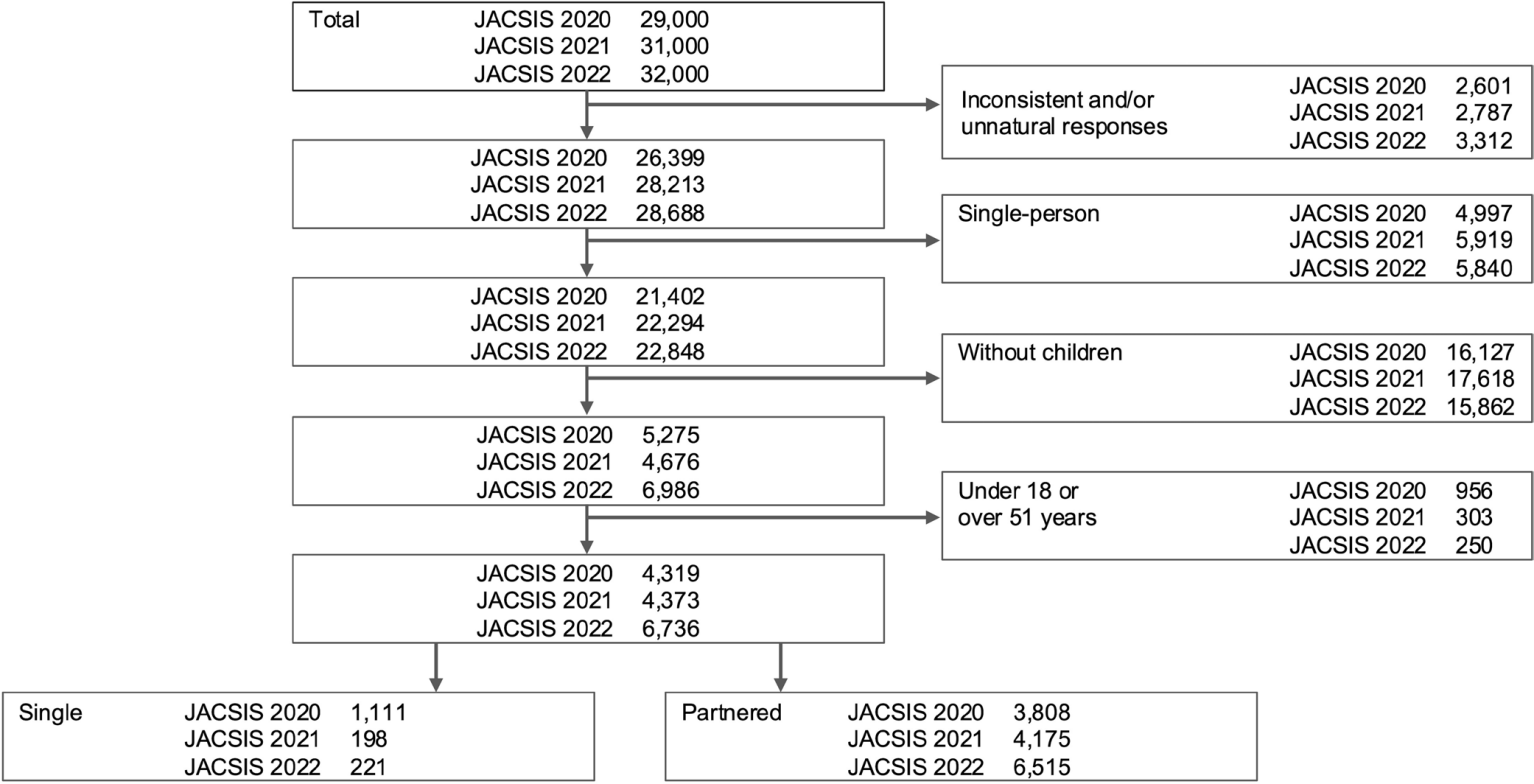
Flowchart of the participants. JACSIS, Japan COVID‐19 and Society Internet Survey.

The final analysis included 16,028 participants (4919, 4373, and 6736 from the 2020, 2021, and 2022 surveys, respectively; Figure 1). Table 1 presents the demographic and socioeconomic characteristics of the participants. Single parents accounted for 1530 (200 single fathers, 1330 single mothers). Aggregated across the three survey years, single parents were more likely to live in three‐generation households, report lower annual household incomes (under 3 million JPY), and have nonregular employment than partnered parents. In particular, single mothers were disproportionately represented in the low‐income category (52%, 566/1092) compared with single fathers (14%, 24/173). Additionally, chronic conditions were more prevalent among single parents than partnered ones. Across all years and stratifications, single parents consistently exhibited a higher prevalence of both psychological distress (K6 ≥ 5) and poor SRH (very poor or poor) than their partnered counterparts. Between single fathers and mothers, psychological distress was potentially more prevalent among single fathers (51%, 101/200 vs. 42%, 554/1330).

**TABLE 1:** Sociodemographic characteristics of the study participants.

	2020						2021						2022					
	Father (*N* = 2001)		*p*	Mother (*N* = 2918)		*p*	Father (*N* = 2128)		*p*	Mother (*N* = 2245)		*p*	Father (*N* = 2706)		*p*	Mother (*N* = 4030)		*p*
	Single (*N* = 128)	Partnered (*N* = 1873)		Single (*N* = 983)	Partnered (*N* = 1935)		Single (*N* = 36)	Partnered (*N* = 2092)		Single (*N* = 162)	Partnered (*N* = 2083)		Single (*N* = 36)	Partnered (*N* = 2670)		Single (*N* = 185)	Partnered (*N* = 3845)	
Age (years), *n* (%)			< 0.001			0.013			< 0.001			0.003			0.6			< 0.001
15–20	11 (8.6%)	12 (0.6%)		9 (0.9%)	10 (0.5%)		3 (8.3%)	4 (0.2%)		4 (2.5%)	3 (0.1%)		0 (0%)	0 (0%)		1 (0.5%)	2 (< 0.1%)	
21–30	13 (10%)	138 (7.4%)		110 (11%)	280 (14%)		3 (8.3%)	152 (7.3%)		19 (12%)	279 (13%)		4 (11%)	231 (8.7%)		49 (26%)	762 (20%)	
31–40	35 (27%)	814 (43%)		471 (48%)	958 (50%)		17 (47%)	940 (45%)		78 (48%)	972 (47%)		22 (61%)	1492 (56%)		81 (44%)	2272 (59%)	
> 40	69 (54%)	909 (49%)		393 (40%)	687 (36%)		13 (36%)	996 (48%)		61 (38%)	829 (40%)		10 (28%)	947 (35%)		54 (29%)	809 (21%)	
Educational attainment, *n* (%)			< 0.001			< 0.001			0.016			< 0.001			0.01			< 0.001
Junior high school	2 (1.6%)	8 (0.4%)		22 (2.2%)	12 (0.6%)		1 (2.8%)	4 (0.2%)		5 (3.1%)	17 (0.8%)		2 (5.6%)	17 (0.6%)		8 (4.3%)	50 (1.3%)	
High school/vocational school	55 (43%)	524 (28%)		541 (55%)	755 (39%)		12 (33%)	564 (27%)		84 (52%)	769 (37%)		12 (33%)	726 (27%)		99 (54%)	1303 (34%)	
University/junior college/technical college	71 (55%)	1338 (71%)		417 (42%)	1165 (60%)		22 (61%)	1515 (72%)		69 (43%)	1291 (62%)		21 (58%)	1910 (72%)		75 (41%)	2475 (64%)	
Other	0 (0%)	3 (0.2%)		3 (0.3%)	3 (0.2%)		1 (2.8%)	9 (0.4%)		4 (2.5%)	6 (0.3%)		1 (2.8%)	17 (0.6%)		3 (1.6%)	17 (0.4%)	
Number of children, *n* (%)			< 0.001			< 0.001			> 0.9			< 0.001			0.2			< 0.001
1	87 (68%)	857 (46%)		732 (74%)	988 (51%)		18 (50%)	992 (47%)		124 (77%)	1078 (52%)		21 (58%)	1260 (47%)		150 (81%)	1949 (51%)	
2	34 (27%)	825 (44%)		207 (21%)	751 (39%)		16 (44%)	889 (42%)		32 (20%)	803 (39%)		13 (36%)	1117 (42%)		26 (14%)	1473 (38%)	
3	2 (1.6%)	166 (8.9%)		39 (4.0%)	175 (9.0%)		2 (5.6%)	184 (8.8%)		6 (3.7%)	182 (8.7%)		1 (2.8%)	259 (9.7%)		8 (4.3%)	369 (9.6%)	
≥ 4	5 (3.9%)	25 (1.3%)		5 (0.5%)	21 (1.1%)		0 (0%)	27 (1.3%)		0 (0%)	20 (1.0%)		1 (2.8%)	34 (1.3%)		1 (0.5%)	54 (1.4%)	
Household structure, *n* (%)																		
Three‐generation	54 (42%)	158 (8.4%)	< 0.001	341 (35%)	129 (6.7%)	< 0.001	6 (17%)	130 (6.2%)	0.024	47 (29%)	109 (5.2%)	< 0.001	9 (25%)	138 (5.2%)	< 0.001	68 (37%)	163 (4.2%)	< 0.001
Nuclear	74 (58%)	1715 (92%)		642 (65%)	1806 (93%)		30 (83%)	1962 (94%)		115 (71%)	1974 (95%)		27 (75%)	2532 (95%)		117 (63%)	3682 (96%)	
Household income (million JPY), *n* (%)			< 0.001			< 0.001			0.11			< 0.001			0.001			< 0.001
< 3	16 (15%)	63 (3.6%)		433 (53%)	135 (8.3%)		3 (9.4%)	57 (3.0%)		61 (48%)	116 (6.9%)		5 (16%)	70 (2.8%)		72 (49%)	200 (6.2%)	
3–10	83 (75%)	1404 (80%)		353 (43%)	1324 (82%)		25 (78%)	1553 (80%)		61 (48%)	1360 (81%)		24 (77%)	1924 (78%)		68 (46%)	2596 (81%)	
> 10	11 (10%)	280 (16%)		31 (3.8%)	160 (9.9%)		4 (13%)	321 (17%)		6 (4.7%)	201 (12%)		2 (6.5%)	471 (19%)		7 (4.8%)	407 (13%)	
No response/Unknown	18	126		166	316		4	161		34	406		5	205		38	642	
Occupation, *n* (%)			< 0.001			< 0.001			< 0.001			< 0.001			0.014			< 0.001
Unemployed	17 (13%)	20 (1.1%)		76 (7.7%)	818 (42%)		4 (11%)	15 (0.7%)		15 (9.3%)	822 (39%)		2 (5.6%)	16 (0.6%)		22 (12%)	1472 (38%)	
Nonregular employee	8 (6.3%)	32 (1.7%)		365 (37%)	562 (29%)		3 (8.3%)	33 (1.6%)		57 (35%)	653 (31%)		1 (2.8%)	42 (1.6%)		62 (34%)	912 (24%)	
Full‐time employee	82 (64%)	1602 (86%)		474 (48%)	490 (25%)		22 (61%)	1795 (86%)		75 (46%)	527 (25%)		26 (72%)	2305 (86%)		88 (48%)	1292 (34%)	
Self‐employed	12 (9.4%)	99 (5.3%)		47 (4.8%)	52 (2.7%)		3 (8.3%)	105 (5.0%)		11 (6.8%)	57 (2.7%)		3 (8.3%)	120 (4.5%)		9 (4.9%)	110 (2.9%)	
Employer	9 (7.0%)	120 (6.4%)		21 (2.1%)	13 (0.7%)		4 (11%)	144 (6.9%)		4 (2.5%)	24 (1.2%)		4 (11%)	187 (7.0%)		4 (2.2%)	59 (1.5%)	
Working hours, *n* (%)			< 0.001			< 0.001			0.021			< 0.001			< 0.001			< 0.001
< 20	29 (23%)	124 (6.6%)		213 (22%)	1253 (65%)		3 (8.3%)	47 (2.2%)		27 (17%)	1143 (55%)		6 (17%)	143 (5.4%)		46 (25%)	2002 (52%)	
20–40	34 (27%)	335 (18%)		385 (39%)	407 (21%)		10 (28%)	343 (16%)		64 (40%)	582 (28%)		9 (25%)	244 (9.1%)		59 (32%)	1032 (27%)	
40–60	55 (43%)	1222 (65%)		359 (37%)	254 (13%)		19 (53%)	1472 (70%)		65 (40%)	320 (15%)		21 (58%)	2027 (76%)		80 (43%)	785 (20%)	
> 60	10 (7.8%)	192 (10%)		26 (2.6%)	21 (1.1%)		4 (11%)	230 (11%)		6 (3.7%)	38 (1.8%)		0 (0%)	256 (9.6%)		0 (0%)	26 (0.7%)	
Medical conditions																		
Physical illness, *n* (%)	30 (23%)	222 (12%)	< 0.001	108 (11%)	148 (7.6%)	0.003	17 (47%)	413 (20%)	< 0.001	40 (25%)	263 (13%)	< 0.001	11 (31%)	475 (18%)	0.047	41 (22%)	442 (11%)	< 0.001
Cancer, *n* (%)	4 (3.1%)	16 (0.9%)	0.035	8 (0.8%)	11 (0.6%)	0.4	2 (5.6%)	24 (1.1%)	0.07	4 (2.5%)	20 (1.0%)	0.089	1 (2.8%)	37 (1.4%)	0.4	3 (1.6%)	23 (0.6%)	0.11
Depression, *n* (%)	6 (4.7%)	46 (2.5%)	0.14	39 (4.0%)	26 (1.3%)	< 0.001	5 (14%)	70 (3.3%)	0.008	5 (3.1%)	32 (1.5%)	0.2	3 (8.3%)	82 (3.1%)	0.1	4 (2.2%)	53 (1.4%)	0.3
Other mental disorder, *n* (%)	9 (7.0%)	38 (2.0%)	0.002	36 (3.7%)	37 (1.9%)	0.004	6 (17%)	69 (3.3%)	0.001	12 (7.4%)	52 (2.5%)	0.002	4 (11%)	69 (2.6%)	0.015	8 (4.3%)	78 (2.0%)	0.059
K6, *n* (%)			0.001			< 0.001			< 0.001			0.045			0.2			< 0.001
No (0–4)	71 (55%)	1317 (70%)		601 (61%)	1349 (70%)		11 (31%)	1233 (59%)		86 (53%)	1290 (62%)		17 (47%)	1646 (62%)		89 (48%)	2426 (63%)	
Moderate (5–12)	37 (29%)	394 (21%)		265 (27%)	443 (23%)		16 (44%)	662 (32%)		55 (34%)	615 (30%)		14 (39%)	728 (27%)		69 (37%)	1074 (28%)	
High (13–24)	20 (16%)	162 (8.6%)		117 (12%)	143 (7.4%)		9 (25%)	197 (9.4%)		21 (13%)	178 (8.5%)		5 (14%)	296 (11%)		27 (15%)	345 (9.0%)	
Self‐rated health^a^, *n* (%)			< 0.001			< 0.001			0.4			0.008			< 0.001			0.001
Not poor	101 (79%)	1678 (90%)		819 (83%)	1748 (90%)		31 (86%)	1875 (90%)		133 (82%)	1853 (89%)		24 (67%)	2344 (88%)		152 (82%)	3450 (90%)	
Poor	27 (21%)	195 (10%)		164 (17%)	187 (9.7%)		5 (14%)	217 (10%)		29 (18%)	230 (11%)		12 (33%)	326 (12%)		33 (18%)	395 (10%)	

*Note:* Categorical variables between the groups were compared using the chi‐squared test. K6, six‐item Kessler Psychological Distress Scale.

^a^ Not Poor means “Excellent,” “Good,” or “Fair”; Poor means “Poor” or “Very Poor”.

**FIGURE 2:**
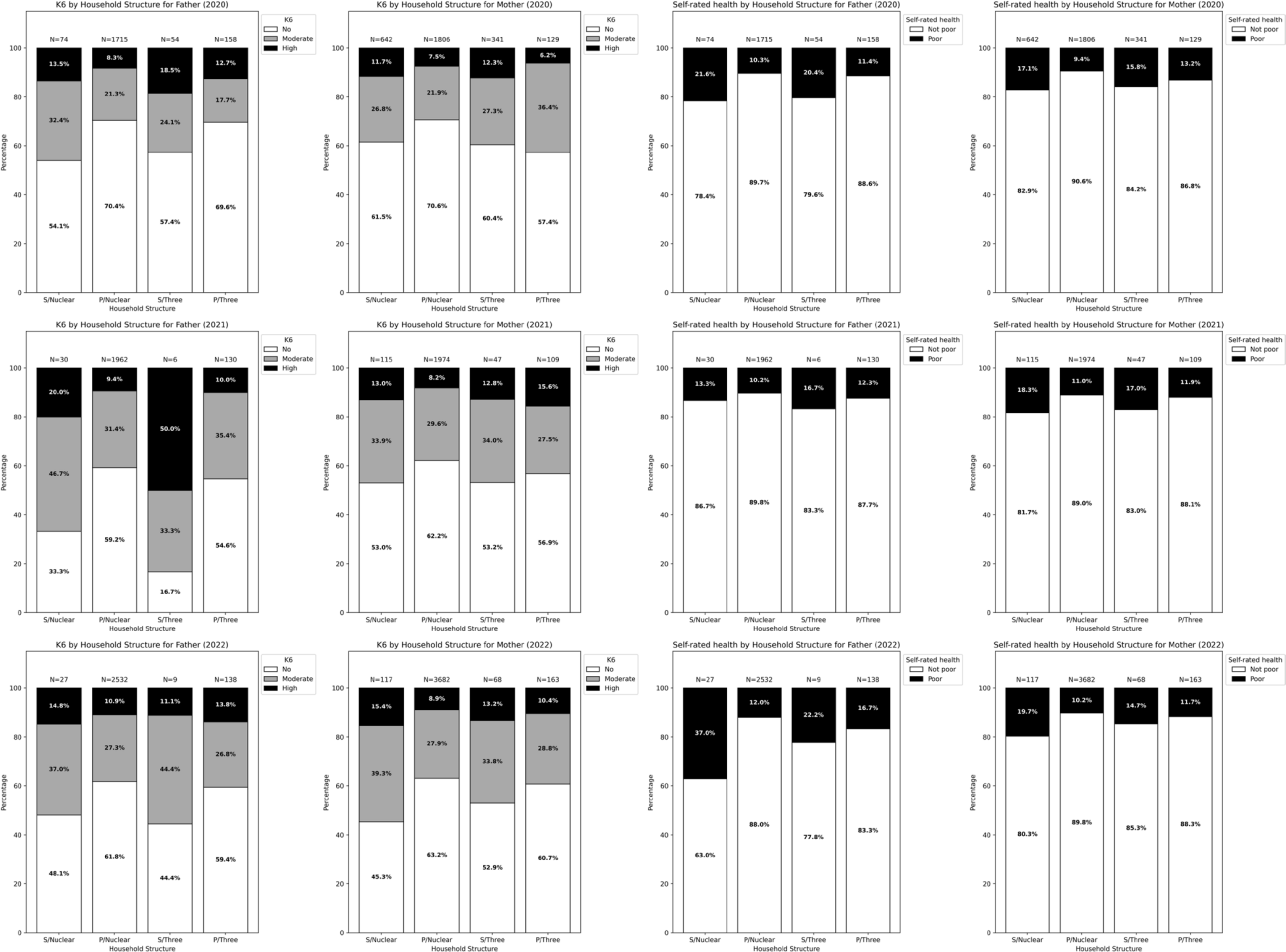
Health outcomes by household structure. Nuclear, nuclear household; P, partnered parent; S, single parent; three, three‐generation household.

In terms of household structure, single‐parent households had a higher prevalence of psychological distress and poor SRH than partnered‐parent households in all years (Figure 2). Aggregated across the three survey years, the prevalence of psychological distress among single fathers was similar regardless of household structure (nuclear: 52%, 68/131; three‐generation: 48%, 33/69), as was the prevalence of poor SRH (nuclear: 23%, 30/131; three‐generation: 20%, 14/69). Likewise, single mothers exhibited comparable rates of psychological distress (nuclear: 42%, 365/874; three‐generation: 41%, 189/456) and poor SRH (nuclear: 18%, 154/874; three‐generation: 16%, 72/456) between such households. Partnered parents also demonstrated a similar lack of difference by household structure for these outcomes.

**FIGURE 3:**
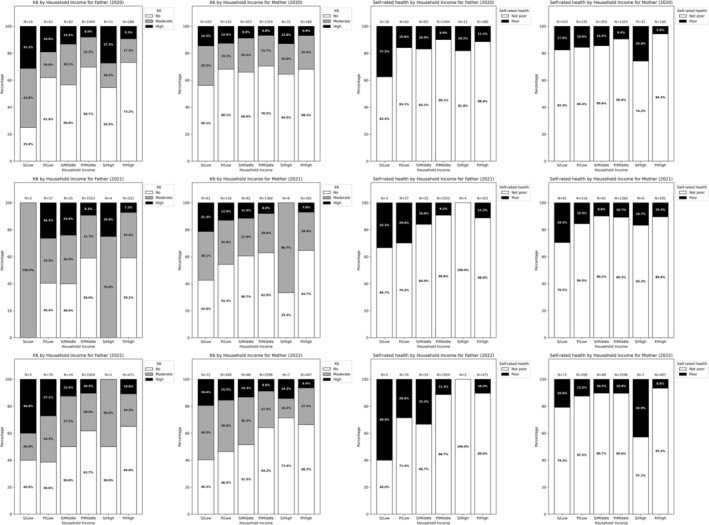
Health outcomes by household income. High, > 10 million JPY; low, < 3 million JPY; middle, 3–10 million JPY; P, partnered parent; S, single parent.

The group with a low annual household income tended to have a higher prevalence of psychological distress than the counterpart (Figure 3). This trend was consistent for both single and partnered parents. Aggregated across the three survey years, the prevalence of psychological distress among single fathers and mothers with a low annual household income was 25% (6/24) and 47% (268/566), and that of poor SRH was 42% (10/24) and 19% (109/566), respectively.

Regardless of occupation (Figure S1) or working hours (Figure S2), psychological distress and poor SRH remained consistently higher among single parents. No specific trend by occupation type or working hours in relation to SRH was observed.

**FIGURE 4:**
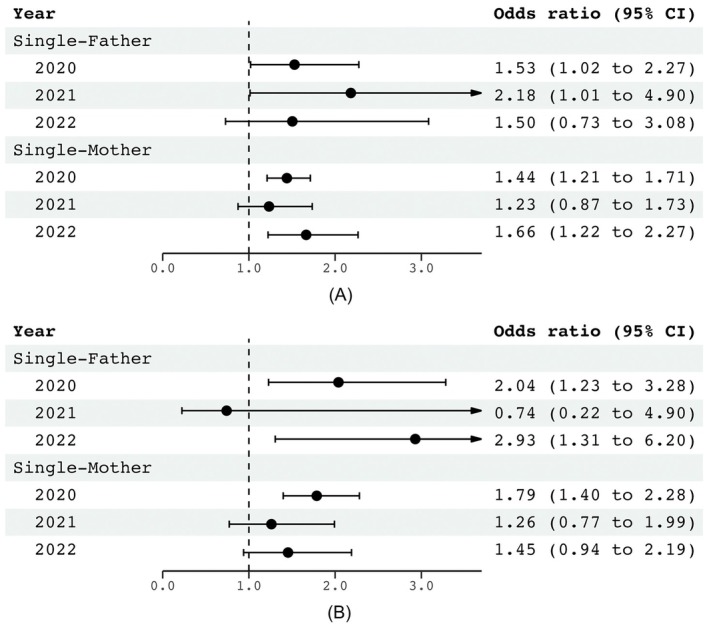
Associations of family type (single or partnered parent) with psychological distress and self‐rated health. Associations between family type (single‐parent or partnered‐parent) and health outcomes were examined using the logistic regression analysis, adjusting for key covariates including age, educational attainment, number of children, and presence of each chronic disease (cancer, noncancer physical illness, depression, and other mental disorders). Psychological distress was dichotomized using a cutoff score of ≥ 5 (low distress vs. moderate‐to‐high distress). (A) K6 and (B) self‐rated health. CI, confidence interval.

Separate logistic regression analyses for each year (2020–2022) showed that single‐parent status was associated with higher odds of psychological distress compared with partnered‐parent status (Figure 4). The adjusted odds ratios (aORs) obtained across these three analyses were 1.50–2.18 for single fathers and 1.23–1.66 for single mothers. Similar associations were found for poor SRH, with aORs of 0.74–2.93 and 1.26–1.79 for single fathers and mothers, respectively.

Regarding longitudinal trends analyzed in 1282 individuals who completed all three surveys, the prevalence of psychological distress increased in 2021 and 2022 compared with that in 2020 (Table S1). Conversely, the prevalence of poor SRH remained relatively stable over time.

## 4 Discussion

The present study, based on large‐scale data from JACSIS across three pandemic years (2020–2022), supported our primary hypotheses. We demonstrated that in Japan, single parents experienced a disproportionately higher burden of psychological distress and physical problems than partnered ones. These disparities persisted when stratified by household structure, household income level, occupation, and working hours, highlighting the structural vulnerability of single‐parent households.

Consistent with our findings, prepandemic studies [16, 17] and studies conducted in countries other than Japan [13, 14, 25] identified single parenthood as an important risk factor for adverse mental and physical health outcomes. The COVID‐19 pandemic likely exacerbated preexisting challenges commonly faced by single parents; these challenges include financial strain, social isolation, difficulties balancing employment and childcare, and limited access to social support networks [26, 27]. The reported odds ratios reflect the overall association between single‐parent status and health, incorporating the effects of these mediating factors rather than direct effects independent of them. Additionally, our post hoc exploratory longitudinal subset analysis hinted at a potential worsening of psychological distress over the pandemic years, suggesting a cumulative burden during the prolonged crisis; nonetheless, this trend requires further research for confirmation.

Notably, given the relative paucity of research specifically addressing single fathers [7, 10, 12, 16], our findings are particularly valuable because they illuminate the often overlooked difficulties encountered by this group. Previous prepandemic studies suggested that single mothers are more psychologically stressed than single fathers [10, 12], but the results of the present study are different. The high level of psychological distress among single fathers during the pandemic underscores the need for further research and adequate support strategies for this population.

In our study, economic hardship appears to function as a key mediator contributing to these observed health disparities. Single parents, especially single mothers, were disproportionately represented in the low‐income category (< 3 million JPY), and they consistently exhibited poorer health outcomes. Consistent with our findings, previous prepandemic research reported a higher prevalence of adverse mental health outcomes among single parents experiencing financial hardships [10, 27]. As highlighted in the Introduction, Japan has high poverty rates among single mothers despite their high employment rates [15, 28]. Our data reflect this structural vulnerability, revealing that economic hardship is a critical mediator of health outcomes. Thus, policies aimed at mitigating economic instability, such as enhanced financial assistance and subsidies for essential needs, may be crucial for improving the health and well‐being of single‐parent households, particularly given that single mothers exhibit heightened economic vulnerability.

Furthermore, as anticipated in our exploratory hypothesis, our study revealed that residing in a three‐generation household conferred no apparent protective effect against mental and physical problems, contrary to previous prepandemic study results [15, 17]. This finding suggests that the potential benefits of multigenerational cohabitation are context‐dependent. During the public health crisis, heightened concerns about intrahousehold virus transmission [29] or increased caregiving responsibilities likely offset these benefits. Therefore, the potential benefits of multigenerational cohabitation may be context‐dependent and might not effectively mitigate the unique stressors encountered during a pandemic. However, in another study, Japanese single mothers during the COVID‐19 pandemic received childcare and financial support from their own parents and siblings, thereby reducing their emotional burden [18]. A limitation of our study is its inability to differentiate whether the co‐residing older generation included participants' own parents or parents‐in‐law. Lacking such information may obscure the protective effect of three‐generation families; hence. caution should be exercised when interpreting the results.

Furthermore, the elevated health risks among single parents persisted even after stratifying by occupation and working hours. Specific employment characteristics and poor health showed no trends in either parent group; thus, the overall burden of balancing work and single‐parent child care was not negated. According to a previous study, single mothers experienced more parenting stress than working mothers with a partner during the pandemic [30]. The complex interplay between employment conditions, family structure, and health warrants further detailed investigation.

This study possesses several strengths. We used a large nationwide dataset covering multiple years of the pandemic, allowing us to examine trends. Additionally, single fathers and single mothers were separately analyzed, providing a more comprehensive picture than studies focusing solely on single mothers.

This study also has some limitations. First, its reliance on a repeated cross‐sectional design prevents the inference of causality, implying that the observed findings merely represent associations. While the dataset included some longitudinal components, the analyses presented are largely cross‐sectional. Furthermore, prepandemic data were not included; thus, we could not fully assess changes from a pre‐event baseline. Second, using an internet‐based survey may introduce selection bias, potentially underrepresenting individuals without internet access, although JACSIS utilized a large, diverse panel. Recall and social desirability biases may also occur in self‐reported data. Third, our definition of single parents encompassed both widowed and divorced individuals. This limitation represents a potential source of heterogeneity, given that the experiences, stressors, and potential health outcomes associated with these different pathways into single parenthood may vary. However, of note, widowed individuals constituted only a small proportion (3.7%, 56/1530) of the single‐parent sample in our study, thereby likely mitigating the overall impact of this heterogeneity on our findings.

In conclusion, this study underscores the persistent mental and physical health disparities faced by single fathers and mothers in Japan during the COVID‐19 pandemic compared with those faced by partnered parents. The findings highlight that economic hardship is an important mediator and suggest that multigenerational households, often considered protective, do not confer the same benefits during the pandemic. Thus, the structural vulnerabilities and socioeconomic disadvantages faced by single‐parent households must be addressed to promote health equity, particularly during and after public health emergencies.

## Author Contributions

**Yasutaka Kuniyoshi:** visualization, writing – original draft, writing – review and editing, methodology, conceptualization, data curation, formal analysis, software, investigation. **Akihito Uezato:** writing – review and editing, supervision, writing – original draft, methodology. **Takahiro Tabuchi:** supervision, writing – review and editing, funding acquisition, conceptualization, data curation, investigation, methodology, software.

## Funding

JACSIS2020 was supported by the Japan Society for the Promotion of Science (JSPS) KAKENHI Grants [grant number 17H03589; 19K10671; 19K10446; 18H03107; 18H03062; 19H03860; 21H04856], the JSPS Grant‐in‐Aid for Scientific Research (KAKENHI) [grant number 19K19439], Research Support Program to Apply the Wisdom of the University to tackle COVID‐19 Related Emergency Problems, University of Tsukuba, and Health Labour Sciences Research Grant [grant number 19FA1005; 19FG2001; 19FA1012] and the Japan Agency for Medical Research and Development (AMED; grant number 2033648). JACSIS2021 was supported by the JSPS KAKENHI Grants (grants 16KK0059, 18H03107, 19K10446, 21H04856); Health Labour Sciences Research Grants (grant number: 19FA1012); Innovative Research Program on Suicide Countermeasures (R3‐2‐2); and Grants from Chiba Foundation for Health Promotion & Disease Prevention. JACSIS2022 was supported by the JSPS KAKENHI Grants (grant number 21H04856; 20K10467; 20K19633; 20K13721), the JST Grant Number JPMJPF201, the Health Labour Sciences Research Grant 21HA2016, the grant for 2021–2022 Strategic Research Promotion (No. SK202116) of Yokohama City University and the research program on “Using Health Metrics to Monitor and Evaluate the Impact of Health Policies,” conducted at the Tokyo Foundation for Policy Research. The findings and conclusions of this article are the sole responsibility of the authors and do not represent the official views of the research funders.

## Ethics Statement

All procedures conformed to the ethical standards of the Helsinki Declaration of 1975, as revised in 2013. Research Ethics Committee of the Osaka International Cancer Institute reviewed and approved the survey protocol (approved on June 19, 2020; approval number: 20084‐2). Additionally, this study was reviewed and approved by the Ethical Committee of the International University of Health and Welfare (No. 24‐TC‐031). The internet survey agency respected the Act on the Protection of Personal Information in Japan.

## Consent

Before responding to the online questionnaire, all participants provided web‐based informed consent.

## Conflicts of Interest

The authors declare no conflicts of interest.

## Supporting information


**Figure S1:** Health outcomes by occupation.

**Figure S2:** Health outcomes by working hours.

**Table S1:** Prevalence of psychological distress and poor self‐rated health by family type among participants completing all three survey waves.

## Data Availability

The data that support the findings of this study are available on request from the corresponding author. The data are not publicly available due to privacy or ethical restrictions.
