# Supplementary material for: Psychological Distress and Self‐Rated Health Among Single Fathers and Mothers During the COVID‐19 Pandemic in Japan
Source: J Gen Fam Med. 2026 May 24;27(3):e70128. doi: 10.1002/jgf2.70128 (PMC13240260; doi:10.1002/jgf2.70128)
Supplement: Supplementary file 3 — Table S1: Prevalence of psychological distress and poor self‐rated health by family type among participants completing all three survey waves. [file JGF2-27-e70128-s001.docx]

**Supplemental Table 1** Prevalence of Psychological Distress and Poor Self-Rated Health by Family Type among Participants Completing All Three Survey Waves

|  | 2020 | 2021 | 2022 | *P*-value |
| --- | --- | --- | --- | --- |
| K6 |  |  |  |  |
| Total (n = 1,282) | 397 (31%) | 527 (41%) | 492 (38%) | <0.001 |
| Single-parents (n = 51) | 20 (39%) | 23 (45%) | 25 (49%) |  |
| Partnered-parents  (n = 1,231) | 377 (31%) | 504 (41%) | 467 (38%) |  |
| Self-related health |  |  |  |  |
| Total (n = 1,282) | 146 (11%) | 140 (11%) | 155 (12%) | 0.45 |
| Single-parents (n = 51) | 10 (20%) | 8 (16%) | 8 (16%) |  |
| Partnered-Parent  (n = 1,231) | 136 (11%) | 132 (11%) | 147 (12%) |  |

K6, six-item Kessler Psychological Distress Scale (K6) (Kessler et al., 2002)

The Cochran’s Q test was used to assess changes in the proportions over the three-year period.

**Supplemental Figure 1 Health outcomes by occupation**S, single parent; P, partnered parent; UE, unemployed; NR, nonregular employee; Full, full-time employee; Self, self-employed; Emp, employer.

**Supplemental Figure 2 Health outcomes by working hours**S, single parent; P, partnered parent; <20, <20 hours per week; 20–40, 20–40 hours per week; 40–60, 40–60 hours per week; ≥ 60, ≥ 60 hours per week
